# Development and implementation of guidelines for the management of depression: a systematic review

**DOI:** 10.2471/BLT.20.251405

**Published:** 2020-08-27

**Authors:** Yena Lee, Elisa Brietzke, Bing Cao, Yan Chen, Outi Linnaranta, Rodrigo B Mansur, Paulina Cortes, Markus Kösters, Amna Majeed, Jocelyn K Tamura, Leanna M W Lui, Maj Vinberg, Jaakko Keinänen, Steve Kisely, Sadiq Naveed, Corrado Barbui, Gary Parker, Mayowa Owolabi, Daisuke Nishi, JungGoo Lee, Manit Srisurapanont, Hartej Gill, Lan Guo, Vicent Balanzá-Martínez, Timo Partonen, Willem A Nolen, Jae-Hon Lee, Ji Hwan Kim, Niels H Chavannes, Tatjana Ewais, Beatriz Atienza-Carbonell, Anna V Silven, Naonori Yasuma, Artyom Gil, Andrey Novikov, Cameron Lacey, Anke Versluis, Sofia von Malortie, Lai Fong Chan, Ahmed Waqas, Marianna Purgato, Jiska Joëlle Aardoom, Josefina T Ly-Uson, Kang Sim, Maria Tuineag, Rianne M J J van der Kleij, Sanne van Luenen, Sirijit Suttajit, Tomas Hajek, Yu Wei Lee, Richard J Porter, Mohammad Alsuwaidan, Joshua D Rosenblat, Arun V Ravindran, Raymond W Lam, Roger S McIntyre, on behalf of the Global Alliance for Chronic Diseases (GACD) Mental Health Guidelines Working Group

**Affiliations:** a Mood Disorders Psychopharmacology Unit, Toronto Western Hospital, University Health Network, 399 Bathurst St 9MP-325, Toronto, Ontario, Canada.; b Department of Psychiatry, Queen's University School of Medicine, Kingston, Canada.; c School of Psychology and Key Laboratory of Cognition and Personality, Southwest University, Chongqing, China.; d Dalla Lana School of Public Health, University of Toronto, Toronto, Canada.; e Bipolar Disorders Clinic, Douglas Mental Health University Institute, Montréal, Canada.; f Pontificia Universidad Católica de Chile, Santiago, Región Metropolitana, Chile.; g Department of Psychiatry II, Ulm University, Ulm, Germany.; h Psychiatric Research Unit, Psychiatric Centre North Zealand, Hilleroed, University Hospital of Copenhagen, Copenhagen, Denmark.; i Department of Public Health Solutions, National Institute for Health and Welfare, Helsinki, Finland.; j School of Clinical Medicine, The University of Queensland, Brisbane, Australia.; k Department of Psychiatry and Behavioral Sciences, University of Kansas Medical Center, Kansas, USA.; l WHO Collaborating Centre for Research and Training in Mental Health and Service Evaluation, Department of Biomedicine and Movement Sciences, Verona, Italy.; m Global Alliance for Chronic Diseases, Wellcome Trust, London, England.; n Center for Genomics and Precision Medicine, University of Ibadan, Ibadan, Nigeria.; o Department of Mental Health, The University of Tokyo, Tokyo, Japan.; p Paik Institute for Clinical Research, Inje University, Busan, Republic of Korea.; q Department of Psychiatry, Chiang Mai University, Chiang Mai, Thailand.; r School of Public Health, Sun Yat-sen University, Guangdong, China.; s Teaching Unit of Psychiatry and Psychological Medicine, University of Valencia, Valencia, CIBERSAM, Spain.; t Department of Psychiatry, University of Groningen, Groningen, Netherlands.; u Department of Psychiatry, Gyeongsang National University Hospital, Jinju, Republic of Korea.; v Department of Public Health and Primary Care, Leiden University Medical Center, Leiden, Netherlands.; w Medical School, University of Valencia, Valencia, Spain.; x WHO European Office for the Prevention and Control of Noncommunicable Diseases, Division of Country Health Programme, Moscow, Russian Federation.; y Psychiatric and Neurological Hospital, Surgut, Russian Federation.; z Maori Indigenous Health Institute, University of Otago, Christchurch, New Zealand.; aa The National Board of Health and Welfare, Stockholm, Sweden.; ab Department of Psychiatry, National University of Malaysia, Kuala Lumpur, Malaysia.; ac Human Development Research Foundation, Islamabad, Pakistan.; ad Department of Psychiatry and Behavioral Medicine, University of the Philippines College of Medicine, Philippines.; ae Department of Mood and Anxiety, Institute of Mental Health, Singapore, Singapore.; af Department of Psychiatry, McGill University, Montréal, Canada.; ag Department of Psychiatry, Dalhousie University, Halifax, Canada.; ah Department of Psychological Medicine, University of Otago, Christchurch, New Zealand.; ai Department of Psychiatry, Faculty of Medicine, Kuwait University, Kuwait.; aj Department of Psychiatry, University of Toronto, Toronto, Canada.; ak Department of Psychiatry, University of British Columbia, Vancouver, Canada.

## Abstract

**Objective:**

To evaluate the development and implementation of clinical practice guidelines for the management of depression globally.

**Methods:**

We conducted a systematic review of existing guidelines for the management of depression in adults with major depressive or bipolar disorder. For each identified guideline, we assessed compliance with measures of guideline development quality (such as transparency in guideline development processes and funding, multidisciplinary author group composition, systematic review of comparative efficacy research) and implementation (such as quality indicators). We compared guidelines from low- and middle-income countries with those from high-income countries.

**Findings:**

We identified 82 national and 13 international clinical practice guidelines from 83 countries in 27 languages. Guideline development processes and funding sources were explicitly specified in a smaller proportion of guidelines from low- and middle-income countries (8/29; 28%) relative to high-income countries (35/58; 60%). Fewer guidelines (2/29; 7%) from low- and middle-income countries, relative to high-income countries (22/58; 38%), were authored by a multidisciplinary development group. A systematic review of comparative effectiveness was conducted in 31% (9/29) of low- and middle-income country guidelines versus 71% (41/58) of high-income country guidelines. Only 10% (3/29) of low- and middle-income country and 19% (11/58) of high-income country guidelines described plans to assess quality indicators or recommendation adherence.

**Conclusion:**

Globally, guideline implementation is inadequately planned, reported and measured. Narrowing disparities in the development and implementation of guidelines in low- and middle-income countries is a priority. Future guidelines should present strategies to implement recommendations and measure feasibility, cost–effectiveness and impact on health outcomes.

## Résumé

**Objectif:**

Évaluer le développement et la mise en œuvre des directives de pratique clinique pour la gestion de la dépression dans le monde.

**Méthodes:**

Nous avons effectué une revue systématique des directives existantes en matière de gestion de la dépression chez les adultes souffrant de troubles dépressifs majeurs ou de troubles bipolaires. Pour chaque directive identifiée, nous avons vérifié le degré de conformité vis-à-vis des mesures de qualité du développement des directives (transparence des processus d'élaboration des directives et de leur financement, composition multidisciplinaire du groupe d'auteurs, revue systématique des études d'efficacité comparative) ainsi que de la mise en œuvre (indicateurs de qualité). Nous avons confronté les directives des pays à faible et moyen revenu avec celles des pays à haut revenu.

**Résultats:**

Nous avons identifié 82 directives de pratique clinique nationales et 13 directives de pratique clinique internationales dans 83 pays et en 27 langues. Les processus d'élaboration des directives et les sources de financement étaient explicitement indiquées dans une moindre proportion chez les pays à faible et moyen revenu (8/29; 28%) par rapport aux pays à haut revenu (35/58; 60%). Le nombre de directives rédigées par un groupe d'auteurs multidisciplinaire était moins élevé dans les pays à faible et moyen revenu (2/29; 7%) que dans les pays à haut revenu (22/58; 38%). Une revue systématique de l'efficacité comparative a été menée pour 31% (9/29) des directives dans les pays à faible et moyen revenu, contre 71% (41/58) des directives dans les pays à haut revenu. Seulement 10% (3/29) des directives émises par les pays à faible et moyen revenu et 19% (11/58) de celles émises par les pays à haut revenu comprenaient un programme d'évaluation des indicateurs de qualité ou de l'adhérence aux recommandations.

**Conclusion:**

La mise en œuvre des directives est mal planifiée, mal analysée et mal mesurée. Réduire les disparités de développement et de mise en œuvre des directives dans les pays à faible et moyen revenu est une priorité. À l'avenir, ces directives devraient prévoir des stratégies d'exécution des recommandations, mais aussi d'évaluation de la faisabilité, de la rentabilité et de l'impact sur l'état de santé.

## Resumen

**Objetivo:**

Evaluar la elaboración e implementación de las directrices de práctica clínica para el tratamiento de la depresión a nivel mundial.

**Métodos:**

Se realizó una revisión sistemática de las directrices existentes para el tratamiento de la depresión en adultos con trastorno depresivo mayor o bipolar. Por cada directriz identificada, se evaluó el cumplimiento de las medidas de calidad de elaboración de directrices (como la transparencia en los procesos de elaboración de directrices y su financiamiento, la composición del grupo multidisciplinario de autores, la revisión sistemática del estudio de eficacia comparativo) y la implementación (como los indicadores de calidad). En este contexto, se compararon las directrices de los países de ingresos bajos y medios con las de los países de ingresos altos.

**Resultados:**

Se identificaron las directrices de práctica clínica a nivel nacional (82) y a nivel internacional (13) de 83 países en 27 idiomas. Los procesos de elaboración de directrices y las fuentes de financiamiento se especificaron de manera explícita en un porcentaje menor de directrices de los países de ingresos bajos y medios (8/29; 28 %) en relación con las de los países de ingresos altos (35/58; 60 %). Un grupo de desarrollo multidisciplinario elaboró menos directrices (2/29; 7 %) para los países de ingresos bajos y medios, en relación con las de los países de ingresos altos (22/58; 38 %). Además, se realizó una revisión sistemática sobre la efectividad comparativa en el 31 % (9/29) de las directrices de los países de ingresos bajos y medios, frente al 71 % (41/58) de las directrices de los países de ingresos altos. Solo el 10 % (3/29) de las directrices de los países de ingresos bajos y medios y el 19 % (11/58) de las directrices de los países de ingresos altos describieron planes para evaluar los indicadores de calidad o el cumplimiento de las recomendaciones.

**Conclusión:**

La implementación de las directrices no se planifica, notifica ni evalúa de manera adecuada a nivel mundial. Es prioritario reducir las desigualdades en la elaboración y la implementación de las directrices en los países de ingresos bajos y medios. Las directrices que se elaboren en el futuro deberán presentar estrategias para implementar las recomendaciones y evaluar la viabilidad, la rentabilidad y el impacto en los resultados sanitarios.

## ملخص

**الغرض:**

تقييم عملية وضع المبادئ التوجيهية للممارسة السريرية للتعامل مع الاكتئاب وتنفيذها على مستوى العالم.

**الطريقة:**

لقد أجرينا مراجعة منهجية للمبادئ التوجيهية الحالية الخاصة بالتعامل مع الاكتئاب لدى البالغين المصابين بحالات الاضطراب الاكتئابي أو الاضطراب ثنائي القطب. وفيما يتعلق بكل مبدأ توجيهي محدد، قمنا بتقييم الامتثال لمقاييس جودة وضع المبدأ التوجيهي (كالشفافية في عمليات وضع المبدأ التوجيهي والتمويل، وتكوين مجموعة من المؤلفين متعددي التخصصات، وكذلك المراجعة المنهجية لأبحاث الفعالية النسبية)، وتنفيذها (مثل مؤشرات الجودة). كما قمنا بمقارنة المبادئ التوجيهية الموجودة في البلدان منخفضة الدخل ومتوسطة الدخل، مع مثيلاتها الموجودة في البلدان مرتفعة الدخل.

**النتائج:**

لقد حددنا 82 مبدأً توجيهيًا وطنيًا، و13 مبدأً توجيهيًا دوليًا من المبادئ التوجيهية الخاصة بالممارسات السريرية، من 83 بلدًا بـ 27 لغة. ولقد تم تحديد عمليات وضع المبدأ التوجيهي، ومصادر التمويل بشكل واضح في نسبة أصغر من المبادئ التوجيهية، الواردة من البلدان منخفضة الدخل ومتوسطة الدخل (8/29؛ 28%)، مقارنة بالبلدان مرتفعة الدخل (35/58؛ 60%). وتم وضع عدد أقل من المبادئ التوجيهية (2/29؛ 7%) الواردة من البلدان منخفضة الدخل ومتوسطة الدخل، بواسطة إحدى مجموعات التطوير متعددة التخصصات، مقارنة بالبلدان مرتفعة الدخل (22/58؛ 38%). وقد تم إجراء مراجعة منهجية للفعالية النسبية في 31% (9/29) من المبادئ التوجيهية للبلدان منخفضة الدخل ومتوسطة الدخل، مقابل 71% (41/58) من المبادئ التوجيهية للبلدان ذات الدخل المرتفع. ولم يتم وصف خطط لتقييم مؤشرات الجودة أو الالتزام بالتوصيات إلا في 10% من المبادئ التوجيهية فقط (3/29) الواردة من البلدان منخفضة الدخل ومتوسطة الدخل، و19% فقط (11/58) من المبادئ التوجيهية للبلدان عالية الدخل.

**الاستنتاج:**

لم يتم التخطيط لعملية تنفيذ المبدأ التوجيهي، والإبلاغ عنها وتقييمها، بشكل مناسب على الصعيد العالمي. إذ يعد تضييق الفوارق في عملية وضع المبادئ التوجيهية وتنفيذها في البلدان منخفضة الدخل ومتوسطة الدخل، إحدى الأولويات. ويجب أن تقدم المبادئ التوجيهية المستقبلية إستراتيجيات لتنفيذ التوصيات، وقياس الجدوى، وفعالية التكلفة، والتأثير على النتائج الصحية. ****

## 摘要

**目的:**

评估全球抑郁症治疗临床实践指南的制定和实施情况。

**方法:**

我们系统评价了现有的成人重度抑郁或双相情感障碍的抑郁治疗指南。针对每份符合纳入标准的指南，我们评估其是否达到指南制定的质量标准（如制定过程和资金的透明度、多学科编著成员、比较疗效研究的系统评价），实施（如质量指标）是否合规。我们对比了中低收入国家和高收入国家的治疗指南。

**结果:**

我们最终确定了 82 份国家级和 13 份国际级的临床实践指南，涉及 83 个国家 27 种语言。与高收入国家相比，中低收入国家指南中明确说明其制定过程和资金来源的比例略低，29 份中仅 8 份给予明确说明，占比 28%，而高收入国家则达 60%，58 份中有 35 份。中低收入国家由多学科编撰成员编著的指南（29 份中仅有 2 份，占比 7%）相较于高收入国家也较少（58 份中有 22 份，占比 38%）。中低收入国家的指南中，有 31% (9/29) 对比较疗效展开系统审查，而高收入国家则达 71% (41/58)。中低收入国家仅有 10% (3/29) 的指南论述了评估质量指标的计划或随附了相关建议，而高收入国家则达 19% (11/58)。

**结论:**

全球范围内，指南的实施均缺乏充分的计划、报道和衡量。缩小中低收入国家在指南制定和实施方面的差距是当务之急。未来制定指南时，应提出实施策略建议，并衡量其可行性、成本效益及对健康结果的影响。

## Резюме

**Цель:**

Оценить разработку и внедрение****практических клинических рекомендаций по лечению депрессии во всем мире.

**Методы:**

Авторы выполнили систематический обзор существующих клинических рекомендаций по лечению депрессии у взрослых с клинической депрессией или биполярным расстройством. Всем идентифицированным клиническим рекомендациям была дана оценка по соответствию критериям качества разработки клинических рекомендаций (таким как прозрачность процессов разработки клинических рекомендаций и финансирования, мультидисциплинарный состав группы авторов, систематический обзор сравнительных исследований эффективности) и реализации (например, показатели качества). Авторы сравнили клинические рекомендации из стран с низким и средним уровнем доходов и из стран с высоким уровнем доходов.

**Результаты:**

Было выявлено 82 национальных и 13 международных клинических рекомендации из 83 стран на 27 языках. Процессы разработки клинических рекомендаций и источники финансирования были четко указаны в меньшем количестве клинических рекомендаций из стран с низким и средним уровнем доходов (8/29; 28%) по сравнению со странами с высоким уровнем доходов (35/58; 60%). Меньшее количество клинических рекомендаций (2/29; 7%) из стран с низким и средним уровнем доходов по сравнению со странами с высоким уровнем доходов (22/58; 38%) было разработано мультидисциплинарной группой авторов. Систематический обзор сравнительной эффективности был выполнен в 31% (9/29) клинических рекомендаций из стран с низким и средним уровнем доходов по сравнению с 71% (41/58) клинических рекомендаций из стран с высоким уровнем доходов. Только в 10% (3/29) клинических рекомендаций из стран с низким и средним уровнем доходов и в 19% (11/58) клинических рекомендаций из стран с высоким уровнем доходов описаны планы по оценке показателей качества или приверженности соблюдению рекомендаций.

**Вывод:**

В глобальном масштабе при внедрении клинических рекомендаций вопросы планирования, отчетности и измерения показателей внедрения решаются неадекватно. Приоритетной задачей является приближение стандартов разработки и внедрения клинических рекомендаций в странах с низким и средним уровнем доходов к таковым в странах с высоким уровнем доходов. В будущих клинических рекомендациях должны быть представлены стратегии по внедрению рекомендаций и оценке их осуществимости, экономической эффективности и воздействию на результаты в отношении здоровья.

## Introduction

The rising prevalence and burden of depression worldwide disproportionately affect low- and middle-income countries.^1^^–^^4^ Major depressive and bipolar disorders independently increase the risk for other chronic diseases, including cardiovascular disease, metabolic syndrome and obesity.^5^^,^^6^ Higher rates of multimorbidity and poorer physical health outcomes are observed among individuals with mental disorders, relative to those without mental disorders; these factors contribute excess morbidity and mortality among individuals with depression, particularly in low- and middle-income countries.^7^^–^^12^ Furthermore, the growing awareness of the social determinants of mental disorders provides the impetus to prioritize the development and implementation of evidence-based practices for depression management in low- and middle-income countries. 

Clinical practice guidelines translate research into recommendations to standardize care, improve health outcomes and reduce morbidity and mortality.^13^^,^^14^ We conducted a systematic review of existing guidelines for the management of depression in adults with major depressive or bipolar disorder. We compared guidelines from low-, middle- and high-income countries to characterize disparities in the development and implementation of guidelines globally.

## Methods

We conducted a systematic review concordant with Preferred Reporting Items for Systematic Reviews and Meta-Analyses recommendations.^15^ Our protocol was registered in the International Prospective Register of Systematic Reviews (CRD42019124759).^16^

### Search strategy

We searched the following online databases from 1994 to January 2019, without language restrictions: Ovid®, MEDLINE® PubMed®, Embase®, ProQuest PsycINFO®; Web of Science, KCI-Korean Journal, Russian Science Citation Index, and SciELO Citation Index; African Journals Online; PakMediNet; EBSCO CINAHL Plus; and Cochrane Library. We searched titles and abstracts using medical search heading terms and keywords. Text keywords used include, for example: bipolar disorder, depressive disorder, mood disorders, depressi*, practice guidelines, evidence-based medicine, guideline*, (medical OR psychiatric association) AND (treatment OR management OR clinical recommendation*). The full search records and details of the grey literature and manual searches are available in the data repository.^17^

### Inclusion and exclusion criteria

We included national and international guidelines for the management of depression in adults (aged approximately 18–70 years) with major depressive or bipolar disorder defined by standardized diagnostic criteria. Diagnostic criteria included the *International statistical classification of diseases and related health problems, *10th edition (ICD-10) and the *Diagnostic and statistical manual of mental disorders* (DSM-IV, DSM-IV-TR and DSM-5). We excluded guidelines published exclusively for the treatment of depressive symptoms in the absence of major depressive or bipolar disorder; developed for use in local regions, hospitals, states or provinces; developed before 1994 (based on, for example, ICD-9 or DSM-III); or with inaccessible full-texts (we approached authors for access to full-text publications of relevant guidelines). Guidelines with original and updated recommendations were considered duplicates (the most recent update was reviewed). Additional selection and data extraction processes are available in the data repository.^17^

### Quality assessment

We evaluated the quality of the guideline development process by assessing compliance to the Institute of Medicine’s eight standards for clinical practice guidelines: (i) transparency in guideline development processes and funding; (ii) disclosure, management and divestment of conflicts of interest; (iii) multidisciplinary and balanced composition of development group; (iv) recommendations based on a systematic review; (v) rating of evidence quality and strength of recommendation grading; (vi) articulation of recommendations; (vii) external review process; and (viii) schedule for guideline update.^14^ A guideline was externally reviewed if it was made available to the general public or target users and relevant stakeholders for comment before its publication. A guideline development group was multidisciplinary and balanced if it included subject-matter experts, clinicians and patient representatives. A guideline met the standard for strength of recommendation grading if all of the following were included for at least three quarters of its recommendations: evidence, harms, benefits, and level of confidence. A guideline clearly articulated its recommendations if each stated recommendation was specific, unambiguous and actionable. 

We adopted measures from the GuideLine Implementability Appraisal and other published criteria to evaluate how amenable each guideline was to implementation.^18^^,^^19^ We assessed characteristics of the guideline development process that facilitate the adoption and application of guideline recommendations: attention to ease of implementation; consideration of economic, legal, social and ethical issues; appraisal of economic or resource implications; evaluation of patient preferences; assessment of implementation enablers and barriers; credibility of authoring individuals or organizations; and the provision of tools to facilitate guideline adoption. We assessed a guideline as having considered the ease of implementation if recommendations requiring minimal resources were presented before those requiring more intensive resources. Less intensive interventions were, for example, those with minimal need for highly skilled personnel, medications, and expensive facilities or infrastructure.^18^ We determined the individuals or organizations who authored the guidelines as having credibility if their expertise was concordant with the target audience (for example, a psychiatric association had published recommendations intended for psychiatrists).

The data extraction form is available in the data repository.^17^

### Critical appraisal

We compared outcome measures between guidelines from high-, upper-middle- and low- or lower-middle-income countries, as classified by the World Bank for the fiscal year of the publication date.^20^ We pooled guidelines from low- and lower-middle-income countries for the analysis as there was only one national guideline from a low-income country. Other low-income countries without national guidelines had guidelines as part of international guidelines. We excluded international guidelines from comparisons between income classifications,^21^^–^^28^ unless they were developed for countries uniformly belonging to a single income classification.^29^^–^^33^

We evaluated to what extent differences in access to quality health care predict disparities in the quality of guideline development processes observed across income classifications. The median Healthcare Access and Quality index was computed for each income classification group using the most recently published index estimates.^34^ The global Healthcare Access and Quality index was 54.4 in 2016; higher indices indicate greater access to quality health care (range: 0–100).^34^

We present numbers and percentages of total number of guidelines across or within income groups, as relevant. We conducted statistical analyses using R software version 3.4.4 (R Foundation for Statistical Computing, Vienna, Austria), with α = 0.05.^35^ We compared outcomes between income classifications using *glm* for logistic regressions. We computed incident rate ratios (IRRs) using msm::*deltamethod* and robust (White–Huber) standard errors (SE) using *sandwich::vcovHC* to evaluate the association between Healthcare Access and Quality index and guideline development quality. 

## Results

**Fig. 1:**
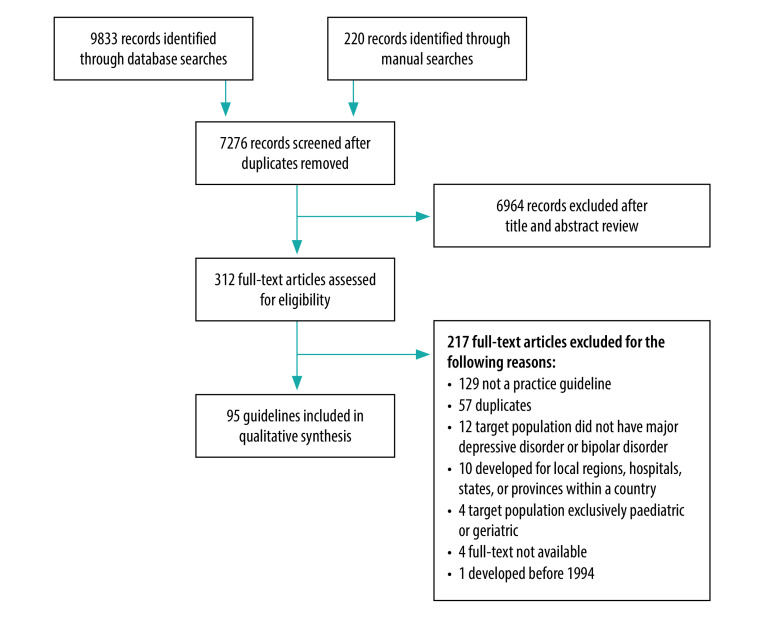
Flowchart of the systematic review of guidelines for management of depression

**Fig. 2:**
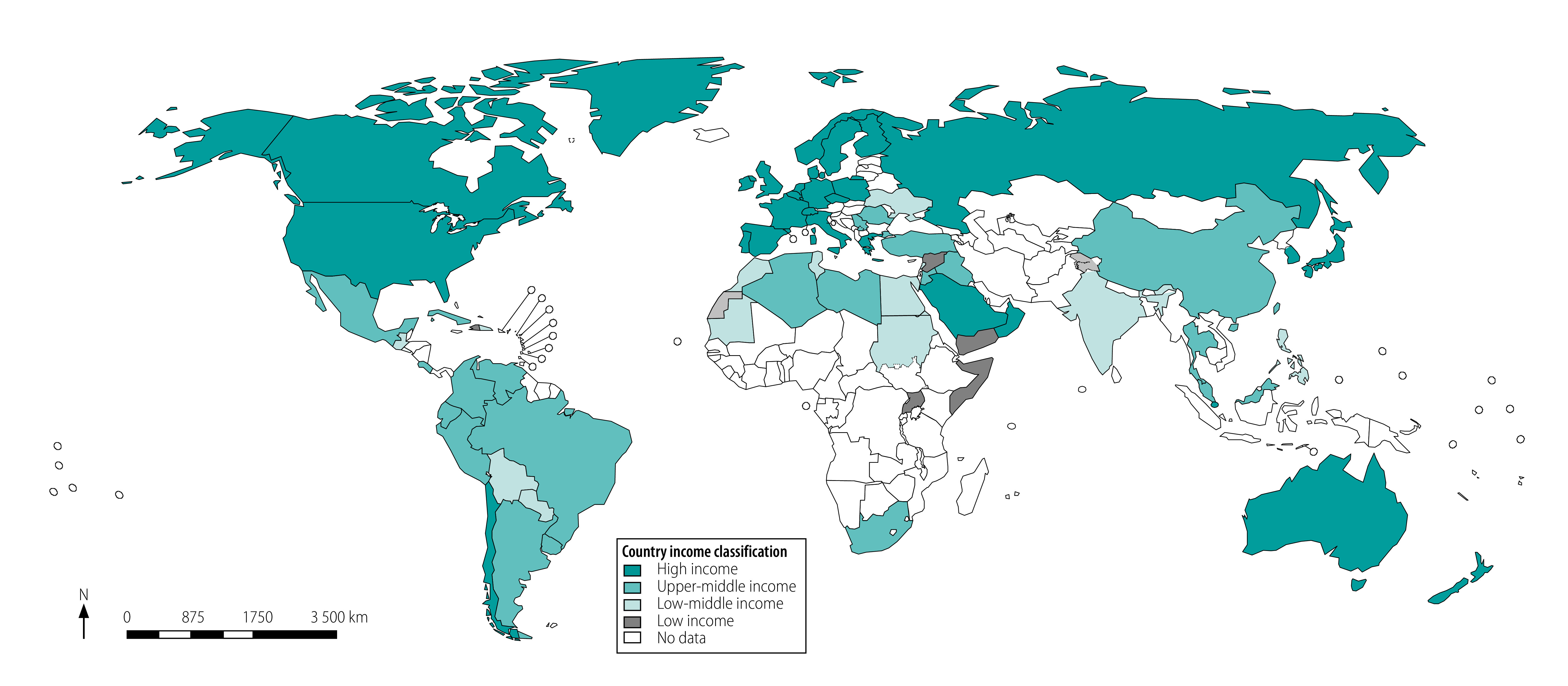
Map of countries with published guidelines for management of depression by country income classification

Notes: Country income groups are World Bank classifications.^20^ We identified 95 guidelines from 83 countries. Countries without national or international guidelines for depression are depicted in white. World Health Organization (WHO) Member States without at least one national or international guideline (other than the WHO guideline) were excluded from the total count of 83 countries and not shaded in the map.

Our database searches yielded 9833 records. After screening the titles and abstracts of non-duplicate records, we reviewed the full texts of 312 records for eligibility (Fig. 1). A total of 95 guidelines from 83 countries met our inclusion criteria (Table 1; available at: http://www.who.int/bulletin/volumes/98/10/20-251405). Fig. 2 (available at: http://www.who.int/bulletin/volumes/98/10/20-251405) illustrates all countries with at least one depression guideline; the countries are grouped by income classification. There were 82 national guidelines^36^^–^^124^ and 13 international guidelines. 

**Table 1:** Guidelines on the management of depression included in the systematic review

Author	Country or territory	Income group^a^	Year	Organization	Scope
American Psychiatric Association, 2002^36^	USA	High	2002	American Psychiatric Association	Bipolar disorder
Ivbijaro, 2005^37^	United Kingdom	High	2004	World Organization of Family Doctors Special Interest Group in Psychiatry & and Neurology	Major depressive disorder
Bauer, 2007^28^	Argentina, Australia, Austria, Belgium, Brazil, Czechia, Denmark, France, Germany, Hungary, Ireland, Italy, Japan, Mexico, Norway, Poland, Republic of Korea, Romania, Russian Federation, Spain, Switzerland, United Arab Emirates, United Kingdom, USA	International	2007	World Federation of Societies of Biological Psychiatry	Major depressive disorder
Ministry of Health of Malaysia, 2007^38^	Malaysia	Upper-middle	2007	Ministry of Health of Malaysia; Ministry of Education of Malaysia, Malaysian Psychiatric Association; Academy of Medicine	Major depressive disorder
Ministry of Health of Sarajevo canton, Institute for Scientific Research & Development, & Clinical Center of the University of Sarajevo, 2007^39^	Bosnia and Herzegovina	Lower-middle	2007	Bosnia and Herzegovina Ministry of Health	Major depressive disorder
Selskab, 2007^40^	Denmark	High	2007	Ministry of Health of Denmark	Major depressive disorder
Latin American Psychiatric Association, 2008^29^	Argentina, Bolivia (Plurinational State of), Brazil, Chile, Colombia, Costa Rica, Cuba, Dominican Republic, Ecuador, El Salvador, Guatemala, Haiti, Mexico, Paraguay, Peru, Puerto Rico, Uruguay, Venezuela (Bolivarian Republic of)	Upper-middle	2008	Latin American Psychiatric Association	Major depressive disorder
National Institute for Health and Care Excellence, 2009^41^	United Kingdom (England, Wales)	High	2009	National Institute for Health and Care Excellence; National Collaborating Centre for Mental Health; British Psychological Society; Royal College of Psychiatrists	Major depressive disorder
Norwegian Medical Association, 2009^42^	Norway	High	2009	Ministry of Health of Norway	Major depressive disorder
American Psychiatric Association, 2010^43^	USA	High	2010	American Psychiatric Association	Major depressive disorder
Federal Government Agency & Mexican Social Insurance Institute, 2010^44^	Mexico	Upper-middle	2010	Ministry of Health of Mexico; Mexican Social Insurance Institute	Bipolar disorder
Grunze et al., 2010^23^	Argentina, Australia, Austria, Belgium, Brazil, Chile, Czechia, Denmark, France, Germany, Hungary, India, Italy, Mexico, Netherlands, Norway, Peru, Poland, Russian Federation, Spain, Switzerland, United Kingdom, USA	International	2010	World Federation of Societies of Biological Psychiatry	Bipolar disorder
Ministry of Public Health of Thailand, 2010^45^	Thailand	Upper-middle	2010	Ministry of Public Health of Thailand	Major depressive disorder
National Institute for Health and Care Excellence, 2009^46^	United Kingdom (England, Wales)	High	2010	National Institute for Health and Care Excellence; National Collaborating Centre for Mental Health; British Psychological Society; Royal College of Psychiatrists	Major depressive disorder with chronic physical health problem
Park et al., 2014^47^	Republic of Korea	High	2010	Korean Neuropsychiatric Association	Major depressive disorder
Scottish Intercollegiate Guidelines Network, 2010^48^	United Kingdom (Scotland)	High	2010	Scottish Intercollegiate Guidelines Network; National Health Service Quality Improvement Scotland	Major depressive disorder
Strejilevich et al., 2010^49^	Argentina	Upper-middle	2010	Argentine Association of Mood Disorders	Bipolar disorder
United States Department of Veterans Affairs, 2010^50^	USA	High	2010	Veterans’ Health Administration, Department of Defense	Bipolar disorder
Brazilian Psychiatric Association, Brazilian Federation of Gynecology and Obstetrics & Brazilian Society of Family and Community Medicine, 2011^51^	Brazil	Upper-middle	2011	Brazilian Psychiatric Association, Brazilian Federation of Gynecology and Obstetrics & Brazilian Society of Family and Community Medicine	Major depressive disorder
Chinese Medicine Association, Chinese Medicine Association Brain Diseases Professional Committee; National Chinese Medicine Administration National Key Encephalology Key Specialist Depression Collaboration Group, 2011^52^	China	Upper-middle	2011	Chinese Medicine Association Brain Diseases Professional Committee, National Chinese Medicine Administration National Key Encephalology Key Specialist Depression Collaboration Group	Major depressive disorder
Mok et al., 2011 ^53^	Singapore	High	2011	Ministry of Health of Singapore	Bipolar disorder
Chua et al., 2012^54^	Singapore	High	2012	Ministry of Health of Singapore	Major depressive disorder
Ministry of Health of Portugal, 2012^55^	Portugal	High	2012	Ministry of Health of Portugal	Bipolar disorder
Ministry of Health of Portugal, 2012^56^	Portugal	High	2012	Ministry of Health of Portugal	Major depressive disorder
Ministry of Health of Serbia, 2012^57^	Serbia	Upper-middle	2012	Ministry of Health of Serbia	Major depressive disorder
Ministry of Health, Social Services and Equality, 2012^58^	Spain	High	2012	Ministry of Health, Social Services and Equality of Spain	Bipolar disorder
Norwegian Medical Association, 2012^59^	Norway	High	2012	Ministry of Health of Norway	Bipolar disorder
Scottish Intercollegiate Guidelines Network, 2012^60^	United Kingdom (Scotland)	High	2012	Scottish Intercollegiate Guidelines Network; National Health Service Quality Improvement Scotland	Peripartum mood and anxiety disorders
Bai et al., 2013^61^	China, Taiwan	High	2013	Taiwanese Society of Biological Psychiatry and Psychopharmacology	Bipolar disorder
Bauer et al., 2013^25^	Argentina, Australia, Austria, Belgium, Brazil, China (Taiwan), Czechia, Denmark, Germany, Hungary, Ireland, Italy, Japan, Mexico, Norway, Republic of Korea, Romania, Russian Federation, Poland, Spain, Switzerland, Turkey, United Arab Emirates, United Kingdom, USA	International	2013	World Federation of Societies of Biological Psychiatry	Major depressive disorder
Begić et al., 2013^62^	Croatia	High	2013	Croatian Psychiatric Association	Major depressive disorder
Chinese Medical Association Society of Neurology, Department of Neuropsychology and Behavioral Neurology; Chinese Medical Association Neurology Branch Parkinson’s Disease and Movement Disorders Group, 2013^63^	China	Upper-middle	2013	Chinese Medical Association Society of Psychiatry, Department of Neuropsychology and Behavioral Neurology; Chinese Medical Association Neurology Branch Parkinson’s Disease and Movement Disorders Group; Chinese Medical Association Neurology Branch, Department of Neuropsychology and Behavioral Neurology	Depressive, anxiety, and psychotic disorders in Parkinson
Federation of Medical Specialists; Dutch Association for Psychiatry, 2013^64^	Netherlands	High	2013	Federation of Medical Specialists; Dutch Association for Psychiatry	Major depressive disorder
Finnish Medical Association Duodecim, Finnish Psychiatric Association; Finnish Society for Adolescent Psychiatry, 2013^65^	Finland	High	2013	Finnish Medical Association Duodecim; Finnish Psychiatric Association; Finnish Society for Adolescent Psychiatry	Bipolar disorder
Gómez-Restrepo et al., 2012^66^	Colombia	Upper-middle	2013	Ministry of Health of Columbia	Major depressive disorder
Grunze et al., 2013^27^	Argentina, Australia, Austria, Chile, Czechia, Belgium, Brazil, Denmark, France, Germany, Hungary, India, Italy, Japan, Mexico, Netherlands, Norway, Peru, Portugal, Russian Federation, Switzerland, Turkey, United Kingdom, USA	International	2013	World Federation of Societies of Biological Psychiatry	Bipolar disorder
Mental Health Directorate, Ministry of Health of Peru, 2013^67^	Peru	Upper-middle	2013	Peru Ministry of Health, Pan American Health Organization	Major depressive disorder
Ministry of Health of Chile, 2013^68^	Chile	High	2013	Ministry of Health of Chile	Major depressive disorder
Ministry of Health of Chile, 2013^69^	Chile	High	2013	Ministry of Health of Chile	Bipolar disorder
Russian Society of Psychiatrists, 2013^70^	Russian Federation	Upper-middle	2013	Russian Society of Psychiatrists	Bipolar disorder
Russian Society of Psychiatrists, 2013^71^	Russian Federation	Upper-middle	2013	Russian Society of Psychiatrists	Major depressive disorder
South African Society of Psychiatrists, 2013^72^	South Africa	Upper-middle	2013	South African Society of Psychiatrists	Psychiatric disorders
Chinese Medical Association Society of Psychiatry, 2014^73^	China	Upper-middle	2014	Chinese Medical Association Society of Psychiatry	Bipolar disorder
Li-Sheng et al., 2014; Chinese Medical Association, Society of Psychiatry^74^	China	Upper-middle	2014	Chinese Medical Association Society of Psychiatry	Major depressive disorder
Czech Psychiatric Society, 2014^75^	Czechia	High	2014	Czech Psychiatric Society	Psychiatric disorders
Kessing et al., 2014^76^	Denmark	High	2014	Ministry of Health of Denmark	Bipolar disorder
Ministry of Health of Malaysia, 2014^77^	Malaysia	Upper-middle	2014	Ministry of Health of Malaysia; Ministry of Education of Malaysia, Malaysian Psychiatric Association; Academy of Medicine	Bipolar disorder
Ministry of Health of Ukraine, 2014^78^	Ukraine	Lower-middle	2014	Ministry of Health of Ukraine; Ukrainian Psychiatric Association	Major depressive disorder
Ministry of Health, Social Services and Equality, 2014^79^	Spain	High	2014	Ministry of Health, Social Services and Equality of Spain	Major depressive disorder
National Institute for Health and Care Excellence, 2014^80^	United Kingdom (England, Wales)	High	2014	National Institute for Health and Care Excellence; National Collaborating Centre for Mental Health; British Psychological Society; Royal College of Psychiatrists	Bipolar disorder
Romanian Society of Psychiatry and Psychotherapy; Romanian Society of Biological Psychiatry and Psychopharmacology, 2014^81^	Romania	Upper-middle	2014	Romanian Society of Psychiatry and Psychotherapy; Romanian Society of Biological Psychiatry and Psychopharmacology	Psychiatric disorders
Samalin et al., 2014^82^	France	High	2014	French Society for Biological Psychiatry and Neuropsychopharmacology	Bipolar disorder
Swedish Psychiatric Association, 2014^83^	Sweden	High	2014	Swedish Psychiatric Association	Bipolar disorder
Bauer et al., 2015^26^	Argentina, Australia, Austria, Belgium, Brazil, China (China, Taiwan), Czechia, Denmark, Germany, Hungary, Italy, Ireland, Japan, Mexico, Norway, Poland, Republic of Korea, Romania, Russian Federation, Spain, Switzerland, Turkey, United Arab Emirates, United Kingdom, USA	International	2015	World Federation of Societies of Biological Psychiatry	Major depressive disorder
Cleare et al., 2015^84^	United Kingdom	High	2015	British Association for Psychopharmacology	Major depressive disorder
Council for the Use of Animal Hospital Medicine, 2015^85^	Denmark	High	2015	Council for the use of Animal Hospital Medicine	Bipolar disorder
Council for the Use of Animal Hospital Medicine, 2015^86^	Denmark	High	2015	Council for the use of Animal Medicine	Major depressive disorder
Dominican Society of Psychiatry, 2015^87^	Dominican Republic	Upper-middle	2015	Dominican Society of Psychiatry	Major depressive disorder
Federal Government Agency & Mexican Social Insurance Institute, 2015^88^	Mexico	Upper-middle	2015	Ministry of Health of Mexico; Mexican Social Insurance Institute	Major depressive disorder
Federation of Medical Specialists; Dutch Association for Psychiatry, 2015^89^	Netherlands	High	2015	Federation of Medical Specialists; Dutch Association for Psychiatry	Bipolar disorder
Malhi et al., 2015^33^	Australia, New Zealand	High	2015	Royal Australian and New Zealand College of Psychiatrists	Major depressive disorder; bipolar disorder
Qaseem et al., 2016^90^	USA	High	2016	American College of Physicians	Major depressive disorder
Danish Health Authority, 2016^91^	Denmark	High	2016	Ministry of Health of Denmark	Major depressive disorder
Finnish Medical Association Duodecim; Finnish Psychiatric Association, 2016^92^	Finland	High	2016	Finnish Medical Association Duodecim; Finnish Psychiatric Association	Major depressive disorder
Goodwin et al., 2016^93^	United Kingdom	High	2016	British Association for Psychopharmacology	Bipolar disorder
Japanese Society of Mood Disorders, 2012^94^	Japan	High	2016	Japanese Society of Mood Disorders	Major depressive disorder
Jobst et al., 2016^31^	Austria, Germany, Hungary, Netherlands, Spain, Sweden, Switzerland, United Kingdom	High	2016	European Psychiatric Association	Major depressive disorder
Kennedy et al. 2016^95^ Lam et al., 2016^96^ Milev et al., 2016^97^ Parikh et al. 2016^98^	Canada	High	2016	Canadian Network for Mood and Anxiety Treatments	Major depressive disorder
Ministry of Health of Uganda, 2016^99^	Uganda	Low	2016	Ministry of Health of Uganda	Medical and psychiatric disorders
Trangle et al., 2016^100^	USA	High	2016	Institute for Clinical Systems Improvement	Major depressive disorder
United States Department of Veterans Affairs, 2016^101^	USA	High	2016	Veterans’ Health Administration, Department of Defense	Major depressive disorder
World Health Organization, 2016^21^	World Health Organization Member States	International	2016	World Health Organization	Mental, neurological, and substance use disorders
Akwa GGZ, 2017^102^^,^^103^	Netherlands	High	2017	GGZ Standards for Dutch Association of Mental Health and Addiction Care	Bipolar disorder
Charpeaud, 2017^104^	France	High	2017	French Society for Biological Psychiatry and Neuropsychopharmacology	Major depressive disorder
Fountoulakis et al., 2017^30^	Austria, Brazil, Canada, China (China, Taiwan), Germany, Israel, Japan, Republic of Korea, Sweden, United Kingdom, USA	High	2017	International College of Neuropharmacology	Bipolar disorder
Gautam et al., 2017^105^	India	Lower-middle	2017	Indian Psychiatric Society	Major depressive disorder
German Society for Bipolar Disorder and German Society of Psychiatry, Psychotherapy and Nervous Diseases, 2018^106^	Germany	High	2017	German Society for Bipolar Disorder; German Society of Psychiatry, Psychotherapy and Nervous Diseases	Major depressive disorder
Grunze et al., 2017^24^	Argentina, Australia, Austria, Canada, Chile, Czechia, Denmark, France, Germany, Hungary, Japan, Netherlands, Poland, Portugal, Romania, Russian Federation, Spain, Switzerland, United Kingdom, USA	International	2017	World Federation of Societies of Biological Psychiatry	Bipolar disorder
Japanese Society of Mood Disorders, 2017^107^	Japan	High	2017	Japanese Society of Mood Disorders	Bipolar disorder
Ministry of Public Health of Ecuador, 2017^108^	Ecuador	Upper-middle	2017	Ministry of Public Health of Ecuador	Major depressive disorder
Okasha et al., 2017^22^	Algeria, Bahrain, Comoros, Djibouti, Egypt, Iraq, Jordan, Kuwait, Lebanon, Libya, Mauritania, Morocco, Oman, Qatar, Saudi Arabia, Somalia, Sudan, Syrian Arab Republic, Tunisia, United Arab Emirates, West Bank and Gaza Strip, Yemen	International	2017	Arab Federation of Psychiatrists	Major depressive disorder
Philippine Psychiatric Association, 2017^109^	Philippines	Lower-middle	2017	Philippine Psychiatric Association	Bipolar disorder
Philippine Psychiatric Association, 2017^110^	Philippines	Lower-middle	2017	Philippine Psychiatric Association	Major depressive disorder
Piotrowski et al., 2017^111^	Poland	High	2017	Polish Psychiatric Association – Wroclaw Division, the Polish Society of Family Medicine and the College of Family Physicians	Major depressive disorder
Seo et al., 2018^112^	Republic of Korea	High	2017	Korean College of Neuropsychopharmacology; Korean Society for Affective Disorders	Major depressive disorder
Shah et al., 2017^113^	India	Lower-middle	2017	Indian Psychiatric Society	Bipolar disorder
Swedish National Board of Health and Welfare, 2017^114^	Sweden	High	2017	Swedish National Board of Health and Welfare	Major depressive disorder
Akwa GGZ, 2018^103^^,^^115^	Netherlands	High	2018	GGZ Standards for Dutch Association of Mental Health and Addiction Care	Major depressive disorder
Chinese Medical Association Chinese Society of Psychiatry, Bipolar Disorder Coordination Group; Chinese Medical Association Psychiatric Branch, 2018^116^	China	Upper-middle	2018	Chinese Medical Association Psychiatric Branch	Bipolar disorder
Japanese Society of Mood Disorders; Japanese Association of Occupational Therapists, 2018^117^	Japan	High	2018	Japanese Society of Mood Disorders; Japanese Association of Occupational Therapists	Major depressive disorder
Woo et al., 2018^118^	Republic of Korea	High	2018	Korean College of Neuropsychopharmacology; Korean Society for Affective Disorders	Bipolar disorder
Yatham et al., 2018^32^	Australia, Brazil, Canada, Japan, Spain, USA	High	2018	Canadian Network for Mood and Anxiety Treatments; International Society for Bipolar Disorders	Bipolar disorder
Dutch General Practitioners Association, 2019^119^	Netherlands	High	2019	GGZ Standards for Dutch Association of Mental Health and Addiction Care	Major depressive disorder
German Society for Bipolar Disorder and German Society of Psychiatry, Psychotherapy and Nervous Diseases, 2019^120^	Germany	High	2019	German Society for Bipolar Disorder; German Society of Psychiatry, Psychotherapy and Nervous Diseases	Bipolar disorder
Samochowiec et al., 2019^121^	Poland	High	2019	Polish Psychiatric Association	Major depressive disorder

^a^ Country income groups are World Bank classifications.^20^ International guidelines are from countries in different income groups.

Of the 13 international guidelines, five were from countries in the same income group.^29^^–^^33^ Of the eight guidelines from countries in different income groups, five covered low- or lower-middle income countries that lacked national guidelines.^21^^–^^24^^,^^28^ However, only two international guidelines included at least one author from a low- or lower-middle-income country.^21^^,^^22^ Guidelines were published in 27 languages, predominantly in the English language (40 guidelines; 42%).

### Target populations and users

Fifty-two guidelines were specifically developed for major depressive disorder and 33 for bipolar disorder. One guideline was developed for the treatment of mood disorders, four for psychiatric disorders, one for psychiatric and neurological disorders, one for medical and psychiatric disorders, and three for depression in special populations (perinatal depression, major depressive disorder with chronic pain). Guidelines most often targeted psychiatrists (77 guidelines; 81%) and primary care providers (65 guidelines; 68%). Only 19 (20%) and 13 (14%) of guidelines targeted policy-makers and payers (companies or organizations that finance the provision of health services), respectively (Table 2).

**Table 2:** Target audience and scope of guidelines for the management of depression, by country-level income classification

Variable	No. (%) of guidelines by income group			
	High income	Upper-middle income	Low- and lower-middle income	International
**Target audience**				
Psychiatrists	52 (90)	15 (68)	6 (86)	4 (50)
Primary care providers	43 (74)	13 (59)	3 (43)	6 (75)
Other specialists	34 (59)	11 (50)	2 (29)	2 (25)
Psychologists	32 (55)	11 (50)	2 (29)	0 (0)
Nurses	31 (53)	9 (41)	2 (29)	1 (13)
Patients	19 (33)	0 (0)	1 (14)	0 (0)
Policy-makers	14 (24)	2 (9)	1 (14)	2 (25)
Payers	9 (16)	3 (14)	1 (14)	0 (0)
**Scope and intent**				
Comorbidities				
Psychiatric	41 (71)	14 (64)	5 (71)	5 (63)
Cardiometabolic	41 (71)	11 (50)	4 (57)	2 (25)
Screening^a^	36 (62)	17 (77)	4 (57)	0 (0)
Primary prevention	10 (17)	4 (18)	2 (29)	0 (0)
Work-related decision support	14 (24)	1 (5)	0 (0)	0 (0)

^a^ The majority of guidelines recommended the use of two diagnostic questions to screen for depressive symptoms: “During the past two weeks, have you often been bothered by little interest or pleasure in doing things” and “During the past two weeks, have you often been bothered by feeling down, depressed or hopeless.”^125^

Notes: Country income groups are World Bank classifications.^20^ International guidelines are from countries in different income groups. Total number of guidelines included: high-income countries: 58; upper-middle-income countries: 22; low- or lower-income countries: 7; international (different income groups): 8.

### Scope and intent

The majority of guidelines recommended the use of the two-item Patient Health Questionnaire^125^ to screen for depressive symptoms. Fifty-seven guidelines (60%) provided recommendations related to depression screening, 51 of which supported screening for depression in the target setting (such as primary care), either systematically or selectively (such as in high-risk populations, postpartum women or settings with resources available for managing depression). The majority of these guidelines recommended the use of the two-item Patient Health Questionnaire^125^ to screen for depressive symptoms. Six guidelines recommended against screening for depression, citing insufficient evidence supporting its effectiveness. Most guidelines with screening recommendations were developed in high- or upper-middle-income countries (53/57 guidelines). The majority of guidelines included recommendations for screening, measuring or treating cardiovascular and metabolic comorbidities (58 guidelines; 61%) or psychiatric comorbidities (65 guidelines; 68%; Table 2).

Recommendations for the primary prevention of depression were included in 16 guidelines (17%), most of which were developed in high-income (10 guidelines) or upper-middle-income countries (four guidelines). These guidelines described risk factors, strategies for reducing risk (such as lifestyle modification, managing stress, psychoeducation or psychosocial support) and methods for early detection. Few guidelines evaluated the literature on the effectiveness of different interventions for primary prevention or cited limitations of current evidence.

Fifteen guidelines (16%) provided decision support or recommendations for assessing work ability, sick leave or return to work; all were published by high-income or upper-middle-income countries (Table 2). Eleven of these guidelines originated in Europe,^42^^,^^58^^,^^59^^,^^65^^,^^92^^,^^114^^,^^120^ while four of these guidelines originated in Canada, Chile, Colombia and Japan.^66^^,^^68^^,^^102^^,^^103^^,^^115^^,^^117^^,^^119^^,^^126^ The recommendations were often limited to the discussion of standardized scales for measuring work-related impairment, factors moderating patients’ return to work, resources for supporting patient employment or occupational rehabilitation and regional disability legislations. The guidelines from Colombia, Finland, Netherlands and Sweden recommended that patients continue to work, unless otherwise indicated, and advised patients and clinicians to discuss work-related factors that may hinder recovery.^66^^,^^92^^,^^102^^,^^103^^,^^114^^,^^115^ Notably, the Swedish bipolar disorder guideline listed an employment rate of 50% among patients as a national target.^122^

### Development processes

The quality of the guideline development processes varied across country income classifications. The median number of standards met was five (interquartile range: 3–7) for high-income country guidelines, two (interquartile range: 1–4) for upper-middle-income country guidelines and one (interquartile range: 0–1.5) for low- or lower-middle-income country guidelines. The World Health Organization (WHO) guidelines, developed specifically for low- and lower-middle-income countries,^21^ met all but one Institute of Medicine-defined standard (systematic review of cost–effectiveness). 

**Fig. 3:**
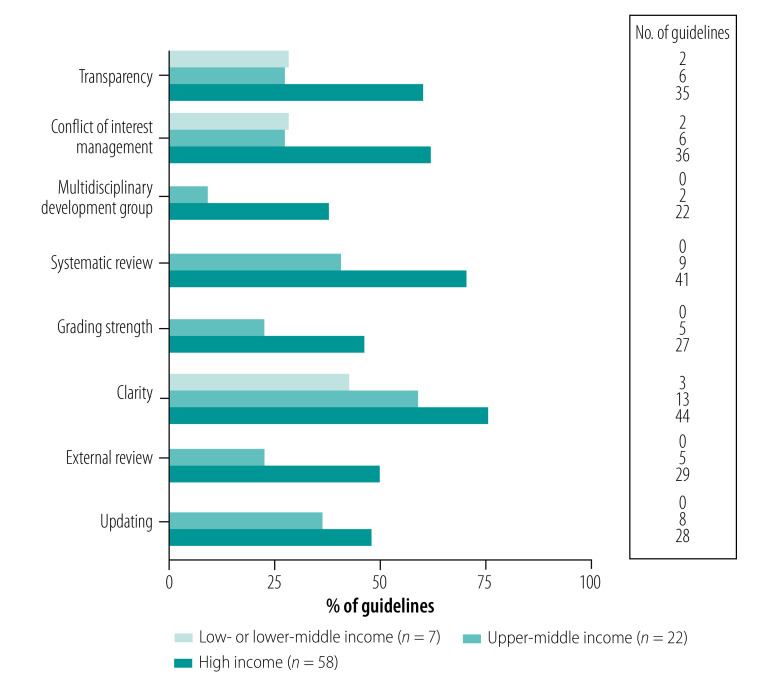
Quality of development process of guidelines for management of depression by country income classification

Notes: Country income groups are World Bank classifications.^20^ Quality of guideline development processes were appraised by measuring compliance to the Institute of Medicine-defined standards for clinical practice guidelines.^14^

Sixty-eight guidelines (72%) provided specific, unambiguous and actionable recommendations, representing 44 of 58 (76%), 13 of 22 (59%) and three of seven (43%) of guidelines from high-, upper-middle- and low- or lower-middle-income countries, respectively (Fig. 3).

The guideline development processes and funding sources were explicitly specified in 51 guidelines (54%), only two of which originated in low- or lower-middle-income countries. Potential conflicts of interest were openly declared and managed in a higher proportion of guidelines from high-income (36 guidelines; 62%) versus upper-middle-income (six guidelines; 27%) and low- or lower-middle-income countries (two guidelines; 29%).

Only 25 guidelines (26%) were developed by a multidisciplinary group comprising subject experts, clinicians and patients or patient advocates. Development groups often lacked patient or patient advocacy representation. None of the low- or lower-middle-income country guidelines had a multidisciplinary development group.

A systematic review of comparative effectiveness of interventions being recommended had been carried out by 57 guidelines (60%), all of which were developed by international authorship groups or in high- or upper-middle-income countries. Some guidelines from low- or middle-income countries were based on recommendations of other published international guidelines. Only 10 guidelines (11%), all from high- and upper-middle-income countries, had conducted a systematic review of cost–effectiveness of a particular intervention or set of recommendations. 

Forty guidelines (42%) included with their recommendations ratings of evidence, harms, benefits, and confidence level. More guidelines from high-income countries (27 guidelines; 47%) met the Institute of Medicine’s standard for strength of recommendation grading. Thirty-five guidelines (37%) had been externally reviewed (for example, by being posted for public comment or reviewed by stakeholders external to the development group); none of these guidelines originated in low- or lower-middle-income countries.

Thirty-eight guidelines (40%) stated plans to renew or update their recommendations, excluding three guidelines that were withdrawn past the scheduled updating date.^36^^,^^54^^,^^78^ Fewer guidelines provided a scheduled date for renewal (26 guidelines; 27%). The scheduled renewal date of these guidelines was often within 3–5 years of the publication date (mean: 4 years; standard deviation; SD: 2). Notwithstanding, 49 guidelines (52%) were revisions, of which 17 guidelines were published within 5 years of the previous iteration. On average, guidelines were revised within 7 years (SD: 3). How frequently and how recently revisions were published were similar between high- and middle-income countries (Fig. 3). None of the guidelines from low- or lower-middle-income countries stated plans to revise recommendations or included a renewal date.

**Fig. 4:**
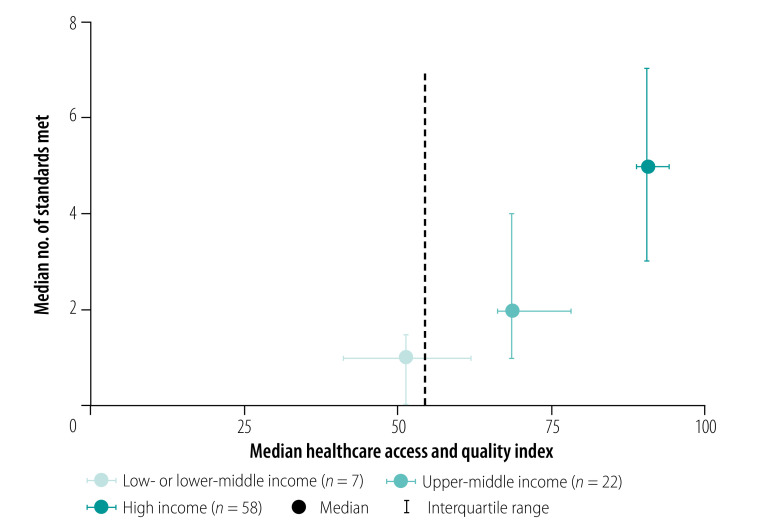
Overall quality of guidelines for management of depression by country income classification

Notes: Country income groups are World Bank classifications.^20^*y*-axis is the median number of Institute of Medicine-defined standards met by guidelines within each income classification group. The dotted line indicates the global Healthcare Access and Quality index of 54.4 in 2016.^34^

The median Healthcare Access and Quality index was significantly greater among guidelines from high-income countries (median: 90.6; interquartile range: 88.8–94.0) relative to those from upper-middle-countries (median: 68.5; interquartile range: 66.3–77.9) and low- or lower-middle-income countries (median: 51.2; interquartile range: 41.2–61.7; *χ^2^* = 156.2, degrees of freedom = 72; *P* < 0.001; Fig. 4). Guidelines from countries with higher Healthcare Access and Quality indices met more Institute of Medicine-defined standards (IRR: 1.03; robust SE: 0.006).

### Facilitators and barriers of implementation

**Fig. 5:**
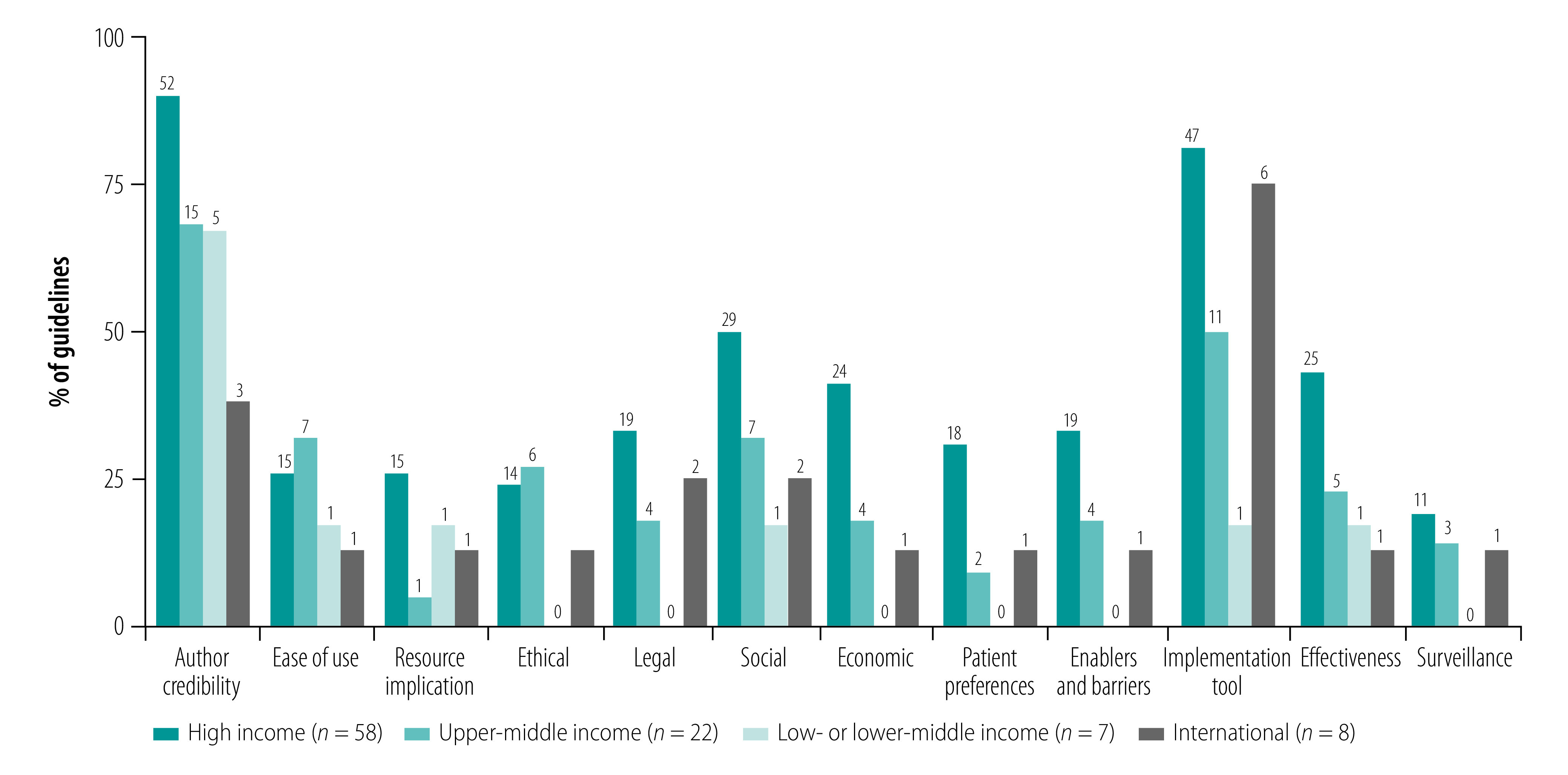
Implementability of guidelines for management of depression by country income classification

Notes: Country income groups are World Bank classifications.^20^ The numerator is given above each bar. International guidelines are from countries in different income groups. We adopted measures from the GuideLine Implementability Appraisal and other published criteria to evaluate how amenable each guideline was to implementation.^18^^,^^19^

The target patient population and intended users were clearly defined in 93 (98%) and 79 (83%) guidelines, respectively. The authors of 75 guidelines (79%) met criteria for credibility with the intended audience (Fig. 5). Most of these guidelines originated in high-income countries (52 guidelines).

Target users or patient representatives evaluated enablers and barriers to the implementation of 24 guidelines (25%); 11 guidelines involved both target users and patient representatives, 11 guidelines involved target users without patient representatives and two guidelines involved only patient representatives in the evaluation of enablers and barriers. None of the guidelines from low- or lower-middle-income countries evaluated enablers and barriers to implementation.

Twenty-one guidelines (22%) evaluated patient preferences by conducting literature reviews of patient preferences or by including patient representatives in the guideline development group, as external reviewers or as members of focus groups. None of the low- or lower-middle-income country guidelines evaluated patient preferences.

Twenty-four guidelines (25%) ordered their recommendations by ease of use (for example, using a stepped-care model). For management of mild depression, these guidelines recommended low-intensity psychosocial and psychological interventions (for example, physical activity, psychoeducation, sleep modification or computerized cognitive behavioural therapy) before pharmacological interventions (for example, selective serotonin reuptake inhibitors) or high-intensity psychological interventions (for example, cognitive behavioural therapy or interpersonal psychotherapy). Whether a guideline had ordered recommendations by ease of use varied across income classifications.

Eighteen guidelines (19%), mostly from high-income countries (15 guidelines), evaluated the resource implications of implementing guideline recommendations. Five guidelines described personnel, infrastructure and training requirements for each recommendation in detail.^21^^,^^41^^,^^66^^,^^80^^,^^114^ Costs and other economic considerations informed the development of 29 guidelines (30%), 24 of which were from high-income countries. Several European guidelines conducted modelling analyses to project the cost–effectiveness and budget impact of their recommendations.^41^^,^^80^^,^^102^^,^^114^^,^^115^^,^^119^

The number of guidelines that considered legal or ethical issues did not vary across income classifications. Twenty-five guidelines (26%) discussed various legal aspects of patient care, such as involuntary treatment of psychiatric patients, certification requirements for professionals providing psychotherapy, availability of antidepressants across national regulatory agencies, national work or disability legislations and statutory patient rights. Twenty-one guidelines (22%) discussed ethical considerations relevant to care provision, such as risks versus benefits of taking medications while pregnant or breastfeeding and obtaining informed patient consent before initiating electroconvulsive therapy or off-label drug usage.

Thirty-nine guidelines (41%) discussed social aspects affecting patient care or illness presentation, such as race or ethnicity, and advised clinicians to consider patient factors, such as social support availability, interpersonal relationship quality, workplace or other factors influencing recovery, childhood trauma and developmental disabilities. Other guidelines, for example, emphasized the importance of adapting guidelines to local contexts and training end-users to be culturally sensitive. Some guidelines commented on the lack of availability of personnel with sufficient training in some areas of the country and the implications of this for clinical care. More guidelines from high-income countries (29 guidelines; 50%) were informed by social considerations when compared to upper-middle-income (seven guidelines; 32%) and low- or lower-middle-income (none) countries.

### Monitoring implementation

Thirty-three guidelines (35%), mostly from high-income countries (25 guidelines), operationalized monitoring or auditing criteria for assessing the implementation of guidelines. These guidelines suggested quality indicators or measures of guideline concordance, such as the proportion of patients prescribed lithium or a selective serotonin reuptake inhibitor for at least four weeks.

Fifteen guidelines (16%), none of which were from low- or lower-middle income countries, described plans for assessing implementation of guidelines or adherence to guideline recommendations (Fig. 4). However, none of these guidelines provided plans to assess whether these actions would improve health or functional outcomes or cost–effectiveness. 

Guidelines described, for example, available health administrative data sets or national electronic medical records that could be used to assess measures of guideline implementation and quality indicators. The Swedish National Quality Register for Bipolar Disorder included longitudinal data from 244 active health-care providers and approximately 30% of patients with bipolar disorder in Sweden.^127^ Quality indicators included the percentages of patients diagnosed with a structured diagnostic instrument, receiving psychoeducation, currently employed or who relapsed with a recurrent mood episode in 12 months, as well as sex and regional differences in lithium prescription.^127^ The National Institute for Health and Care Excellence in England measured the adoption of some recommendations across mental health guidelines, such as the proportion of people with subthreshold or mild-to-moderate depression receiving low-intensity psychosocial interventions.^41^ WHO described the adoption of the Mental Health Gap Action Programme in 18 Member States, with a focus on informing future implementation plans and characterizing implementation enablers and barriers.^128^

Sixty-five guidelines (68%) provided tools for guideline application, such as a quick reference summary. More high-income country (47/58) and international (6/8) guidelines provided implementation tools. Twenty-four guidelines (25%) described plans for disseminating guidelines, 29 of which originated in high-income countries.

## Discussion

We found that many low- and lower-middle-income countries, especially in Africa, lacked published clinical practice guidelines for the management of depression. However, international guidelines exist that cover or specifically target these countries.^21^^–^^24^^,^^28^

While the overarching aim of guidelines is to improve health outcomes and cost–effectiveness, it remains unclear to what extent guidelines for the management of depression are being implemented and improving health outcomes, particularly in low- and lower-middle-income countries.^128^ Most guidelines lacked plans to assess quality indicators or recommendation implementation. We were unable to identify any national guidelines that included government-sanctioned incentives, such as remuneration, for adhering to guideline recommendations or penalties for not implementing recommendations at point-of-care. A notable exception, not included in the present review, is a guideline for adults with mood disorders from Florida, United States of America.^129^ The guideline is integrated into an e-health infrastructure and mandated to be implemented with practitioner concordance monitoring. Government policies that require health-care providers to adhere to recommendations, via health insurance disbursement for example, may facilitate the implementation of guidelines and monitoring of effectiveness.

The disparities in availability, development processes and quality of guidelines underscore an unmet need for decision support in low- and middle-income countries.^1^^,^^130^ Due to limitations in access to resources, health-care personnel in low- and middle-income countries are additionally constrained in their ability to provide timely and appropriate patient care.^34^^,^^131^^,^^132^ Barriers to the application of standard interventions in many low-resource settings include limitations in the availability of interventions (for example, regulatory approval of certain medicines or acquisition costs) and patient access to health-care professionals (for example, specialist fees, rural regions and private versus public clinics). Limitations in the availability of facilities and resources to monitor serum drug levels and liver or renal function (for example, with lithium treatment) may further limit access to treatments in low-resource settings.^18^^,^^131^^–^^135^ Recommendations to implement guidelines must be sufficiently contextualized with relevant ethical, legal, social and economic considerations.^136^^–^^139^

Low- and middle-income countries are differentially affected by multimorbidity, which drastically reduces life expectancy and increases personal, social and economic burden.^8^^,^^12^^,^^140^ Not only is the prevalence of noncommunicable diseases escalating globally, but the risks of infectious diseases have not declined in low- and middle-income countries, further increasing the burden and complexity of managing chronic conditions in these countries.^141^ However, only 50–67% of low- and middle-income country guidelines provided recommendations for the assessment and management of psychiatric or cardiometabolic comorbidities in depression. Future guidelines should provide guidance for screening and managing multimorbidity in adults with depression.

Most guidelines for the management of depression provided tools for the application of guideline recommendations, such as a summary document or a quick-reference guide. However, less than one fifth of the guidelines we identified provided materials for patients; fewer targeted policy-makers or payers. Guideline implementation requires diversity in the engagement of target audiences and stakeholders, as well as realistic and relevant implementation plans.^142^ Future guidelines, therefore, need to be developed collaboratively by a broader collective of stakeholders.^137^

Guideline development groups should include experts in experimental, observational and contextual evidence and knowledge users (such as clinicians and patient advocates).^14^^,^^137^^,^^143^^,^^144^ However, less than one third of guidelines for depression globally included a multidisciplinary development group; in comparison, approximately 64% (36/56) of guidelines for diabetes mellitus and 52% (12/23) of guidelines for hypertension were developed by a multidisciplinary authorship group.^145^^,^^146^ Many guidelines for depression identified in our study were developed without target-user representatives or patient advocates who would be able to provide guidance on the appropriateness, translatability, feasibility and acceptability of guideline recommendations.

Guidelines endeavour to comprehensively review and corroborate knowledge of intervention efficacy, effectiveness, safety and tolerability. Guidelines must also be informed by an evaluation of the determinants, processes and outcomes of implementing evidence-based recommendations.^137^ However, while 60% of guidelines for the management of depression identified herein were based on a systematic review of intervention efficacy and effectiveness literature, only 25% of guidelines evaluated enablers and barriers to implementation. Such gaps in the development processes of existing guidelines may limit the implementation of guidelines for mood disorders.^147^^–^^149^ Future guidelines for the management of depression should involve a combination of international and local collaboration, taking into consideration contextual factors that may facilitate or hinder access to health services or treatments. Contextual factors that may be relevant include, for example, structural or policy aspects of the health-care system, education and training; access to treatment methods for depression; and availability of modern technology.

The main aim of our initiative was not to synthesize a consensual set of implementation measures across low- and middle-income countries. However, lessons learnt from implementation science across other noncommunicable diseases could be a starting point for determining policy and implementation principles for depression management. For example, internet access may be needed to facilitate guideline dissemination, especially in low- and middle-income countries. The integration of technology may also facilitate chronic disease management. The guiding principles include prioritizing the involvement of stakeholder and end-user input in any policy around implementation, identification of those people most at risk, and appraisal of local health-care resources.

The paucity of depression guidelines from low-income countries may reflect limitations in our search strategy (for example, the African Journals Online database primarily includes articles published in English). We were more likely to identify guidelines available online than in print only. To mitigate this possibility, we contacted members of the Global Alliance for Chronic Diseases and members of national psychiatric or other medical associations across geographical and linguistic world regions. Database searches may miss guidelines published as government reports or in formats other than peer-reviewed journal articles or meeting abstracts. To improve the likelihood of detecting such guidelines, we manually searched the websites of multiple national and international medical associations and ministries of health and included experts from 27 countries across all continents in our collaboration. Thus, the possible selection bias in our search is unlikely to confound our findings of differences in guideline quality and development across economic strata. 

Our large number of evaluators may have resulted in differences in data extraction. However, we completed blinded evaluations in duplicate using structured evaluation forms; a third reviewer independently evaluated all forms. In addition, guidelines were evaluated by two or three reviewers who had not been involved in their development.

The focus of our analysis on guidelines may inadequately capture separate implementation studies of guidelines. Future research should primarily evaluate implementation studies of guidelines. We limited our inclusion criteria to national and international guidelines, which may not capture more regional or local differences in guideline development or implementation. Our comparison of guidelines by country-level income classification and Healthcare Access and Quality index did not consider differences in the availability and accessibility of health care within individual countries. Much of the available research informing guideline recommendations has been conducted in high-income countries, with an over-representation of Caucasian groups, often overestimating patient access to expensive medications and specialized care. 

In conclusion, the implementation of guidelines for the management of depression is inadequately planned, reported and measured. As a result, it remains unknown to what extent guidelines are acceptable to patients and other target users; are feasible and cost–effective; and improve health outcomes. Narrowing the disparities in the development and implementation of guidelines, particularly in low- and middle-income countries, is a priority. Refinement of decision support processes in depression is a critical first step towards the aim of reducing morbidity, especially in low- and middle-income countries. Future guidelines should present strategies to implement recommendations and measure feasibility, cost–effectiveness and impact on health outcomes, co-designed by stakeholders and experts with practical (experiential) knowledge from low- and middle-income countries.
